# Patterns of Congenital Malformations and Barriers to Care in Eastern Democratic Republic of Congo

**DOI:** 10.1371/journal.pone.0132362

**Published:** 2015-07-06

**Authors:** Luc Malemo Kalisya, Kavira Nyavandu, Bahati Machumu, Sylvain Kwiratuwe, Peter H. Rej

**Affiliations:** 1 HEAL Africa, 111 Avenue des Ronds Points, Box 319, Goma, Nord Kivu, Democratic Republic of Congo; 2 Department of Anthropology, University of Florida, Turlington Hall, Room 1112, PO 117305, Gainesville, FL, 32611–7305, United States of America; 3 University of Florida Genetics Institute, University of Florida, PO Box 103610, Gainesville, FL, 32610–3610, United States of America; Sun Yat-sen University, CHINA

## Abstract

**Background:**

An increase of congenital anomalies in the eastern Democratic Republic of the Congo (DRC) has been reported. Congenital malformations (CMs) are not uncommon among newborns and, if left untreated, can contribute to increased neonate morbidity and mortality.

**Methods:**

Medical records of all individuals admitted with a diagnosed CM to HEAL Africa Teaching Hospital (Goma, DRC) from 2002 to 2014 (n=1301) were reviewed. Data were analysed using descriptive statistics to summarize chart records, and inferential statistics to investigate significant barriers to earlier treatment.

**Results:**

Since 2012, the number of patients treated each year for CMs has increased by over 200% compared to the average annual number of cases treated from 2002-2011. Though delayed presentation of patients to HEAL Hospital was very obvious, with an average age of 8.2 years. We find that patient age has been significantly decreasing (p=0.037) over time. The average distance separating patients from HEAL Hospital was 178 km, with approximately one third living 350 km or further from the treatment center. Distance is the most significant (p=3.33x10^-6^) barrier to earlier treatment. When controlling for an interaction between gender and the use of mercy funds, we also find that female patients are at a significant (p=1.04x10^-3^) disadvantage to undergo earlier corrective surgery. This disadvantage is further illustrated by our finding that 89% of women and girls, and over 81% of all patients, required mercy funds to cover the cost of surgery in 2014. Lastly, the mortality rate for surgery was low and averaged less than 1.0%.

**Conclusion:**

Despite a formal end to the war in 2009, and an overall increase in individuals undergoing corrective surgery, distance, poverty, and gender are still massive barriers to CM care at HEAL Hospital, Goma, DRC. We find that patients have been successfully treated earlier by HEAL, although the average age of CM correction in 2014 (4.9 years) is still above average for Sub-Saharan Africa. Thus, we advocate for further funding from the National Government and international health agencies to enable continued treatment of CMs in rural residents of the eastern DRC. Distance, the most significant barrier to care can be mitigated by the implementation of additional mobile clinics and the construction of regional surgery centers along with the associated hiring of surgeons trained in CM repair.

## Introduction

Congenital malformations (CMs) can be defined as a physical abnormality, whether genetically or environmentally determined, that is present at time of birth [1]. They can also refer to an abnormality in structure or form that has been present from birth, to up to a few weeks after birth [2]. For example, abdominal malformations are often triggered by maternal insult during the early intrauterine period (3–8 weeks gestation), while the organs are developing [3] and it is argued that intervention and lifestyle changes during the intrauterine period may result in a decreased likelihood of CM formation [4].

Treating congenital anomalies in Africa presents numerous challenges, including the lack of reliable data on incidence, strong traditional belief systems regarding etiology, associated health problems at presentation, as well as late presentation and the lack of a multidisciplinary approach to care [5]. These challenges are often heightened in conflict regions, such as the eastern Democratic Republic of the Congo (DRC), where a significant increase in the annual incidence of CMs was reported recently [6]. Non-attendance to prenatal care, along with the absence of prevention policies and deliveries outside the hospital, may contribute to late consultation and poor prognosis. Taking these challenges into account, the mortality rate associated with CMs in sub-Saharan Africa remains high [7].

CMs significantly increase morbidity and mortality in children living in the tropics, particularly due to malnutrition and infection [8]. As the cost of treatment increases with time, early diagnosis and treatment is even more important. This study was aimed at identifying the patterns of congenital anomalies treated by surgeons from HEAL Africa Teaching Hospital over the last thirteen years and determining the significant barriers to earlier care. This represents the first study to investigate the prevalence and patterns of CM diagnoses in the eastern DRC. We hope to bring attention to these very treatable conditions, promote awareness of treatment options particularly in rural areas, and encourage the development of future research studies on health care access for treatable conditions.

## Methods

### Setting

Over the past decade, the DRC has been the epicenter of one of the most deadly humanitarian crises in recent history [9,10]. Despite an official end to the conflict in 2009, mortality rates remain 70% higher than pre-war levels, and 55% higher than surrounding sub-Saharan African countries [9]. Ongoing threats to human security relate to the continued presence of armed rebels in the area.

HEAL Africa Hospital is a 200 bed facility situated in Goma, eastern DRC. It is the primary surgical center for correcting congenital abnormalities in a region of approximately 6 million people. Goma is the capital of North Kivu Province, an area that was frequently mired in political instability and civil war from 1994–2009. Most patients treated at HEAL Africa are impoverished, and rely on mercy fund programs such as Smile Train, Cure International, Christian Blind Mission (CBM), USAID, AusHEAL, Médecins Sans Frontières (MSF), Churches, etc. to pay for surgical correction of any congenital abnormalities. Furthermore, the National Government has recognized the surgery center at HEAL Africa Hospital, Goma as the National Centre for Clubfoot Care for the entire country. HEAL has well-established community programs, and its network could be utilized for a preventive and surveillance program of birth defects.

### Patients

Medical records of all patients treated at HEAL Africa Hospital for CMs between 2002 and 2014 were studied. Data extracted from records include age, sex, system affected by CM, diagnosis, distance to hospital, payment status, and surgery location. We retrieved complete data for 1301 cases. This study was reviewed and approved by the HEAL Africa Hospital Ethics Committee. Data were de-identified at the point of data extraction from chart records, and all subsequent work was completed with anonymized personal information.

### Statistical analysis

Summary and comparative statistics were computed using R 3.1.2 (R Core Development Team). We used multivariate regression models to determine independent predictors (e.g., distance, payment type, etc.) of our outcome variable (log transformed age at time of treatment) when multiple potential confounding variables were present. A log transformation of our response variable, age, was decided upon following a Box Cox transformation (lambda value of 0.182). We then utilized a stepwise AIC method to identify our best fit model: E{Y} = β_0_+β_1_X_1_+β_2_X_2_+β_3_X_3_+β_4_X_4_+β_5_X_5_+β_6_X_6_+β_7_X_2_X_5_, incorporating diagnosis, payment type, system affected, distance, gender, year, and the interaction between gender and payment type as our predictor variables. The stepwise process yielded an AIC of 774.0, and the model had an adjusted R^2^ value of 0.41.

## Results

### Case series of congenital malformations treated by a single centre

**Fig 1:**
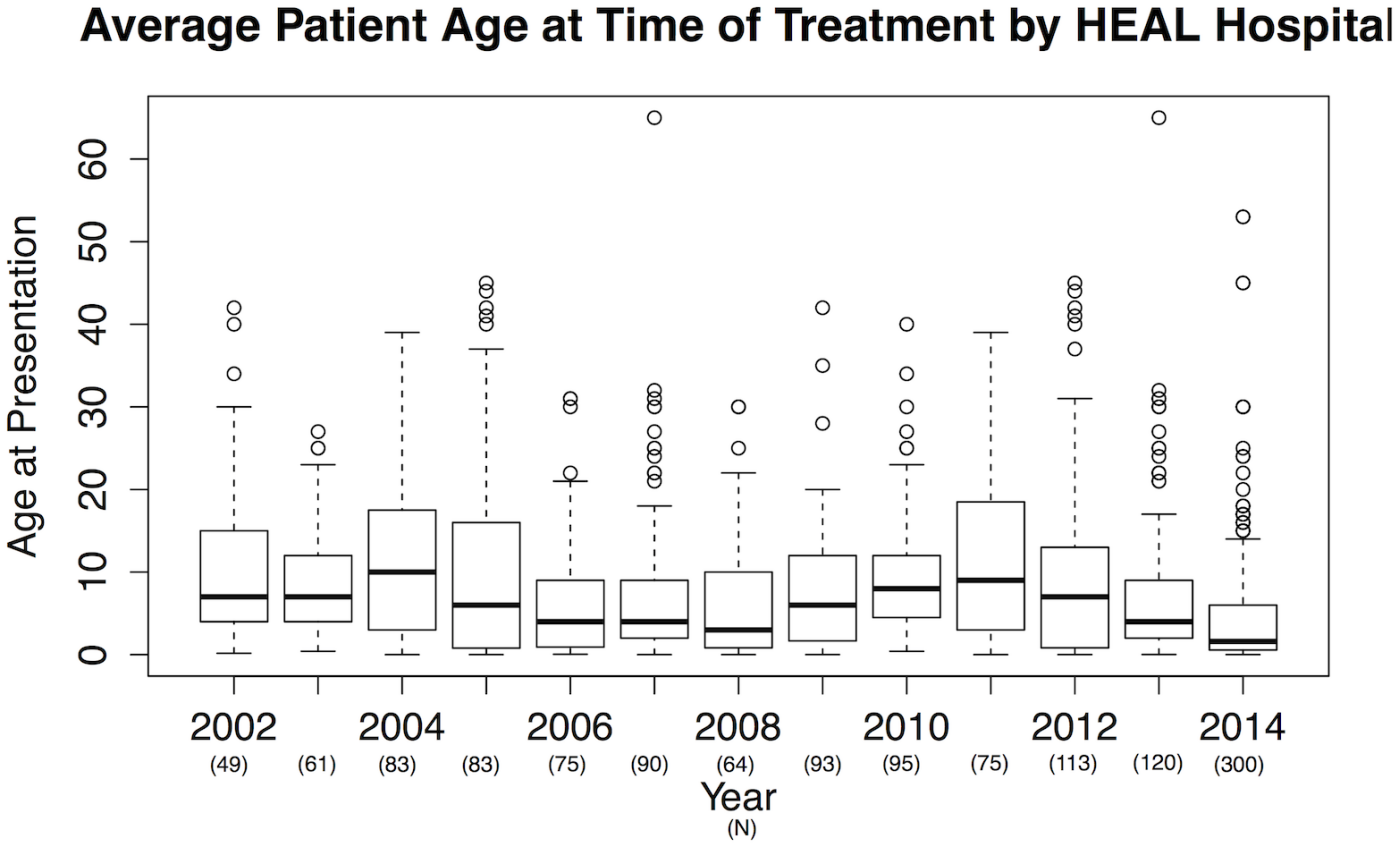
Boxplots depicting the yearly age distributions of patients treated for congenital malformations at HEAL Africa Training Hospital from 2002–2014. The overall average age at time of presentation decreased significantly (p = 0.04) over time, even when controlling for distance from HEAL, payment type, diagnosis, and sex. Boxes indicate interquartile (IQR) variation in age each year; bold lines the median age. Whiskers demonstrate data within the third and quartile +/- 1.5 IQR respectively; dots indicate outliers. Number of individuals treated each year is indicated within parentheses.

We identified 1301 cases of CMs admitted to HEAL Africa Hospital between 2002 and 2014 (Table 1). Delayed presentation was apparent, as the mean patient age was 8.19 (+/- 9.44 SD) with a distribution ranging from 1 day to 65 years (Fig 1). Only 281 cases (21.6%) were corrected before the age of one year, while 419 individuals (32.2%) underwent corrective procedures between the age of 1 and 5 years, and 155 patients (11.9%) were corrected after the age of 17 years. Overall when controlling for all other predictors (barriers), there was a significant decrease in age of treatment over the 13-year study period (p = 0.037).

**Table 1:** Demographics, mean distance to HEAL, payment type, and surgery location of patients treated by HEAL Africa Training Hospital from 2002 to 2014.

**Year presented**	**2002**	**2003**	**2004**	**2005**	**2006**	**2007**	**2008**	**2009**	**2010**	**2011**	**2012**	**2013**	**2014**
**Patients treated (N = 1301)**	49	61	83	83	75	90	64	93	95	75	113	120	300
**Average age when treated (years)**	10.5	9.0	12.2	10.7	6.3	8.3	6.3	7.7	9.8	11.9	9.9	7.8	4.9
**Mean distance from HEAL (km)**	282.7	255.1	197.7	134.3	90.9	70.7	228.4	560.4	289.1	171.5	138.5	62.4	105.5
**N(%) relying on mercy funds**	49 (100%)	61 (100%)	40 (48.2%)	56 (67.5%)	59 (78.7%)	68 (75.6%)	56 (87.5%)	93 (100%)	93 (97.8%)	30 (40.0%)	89 (78.8%)	91 (75.8%)	244 (81.3%)
**N(%) treated by HEAL Mobile Clinic**	49 (100%)	48 (78.7%)	83 (41.0%)	32 (38.6)	13 (17.3%)	18 (20.0%)	41 (64.1%)	93 (100%)	82 (86.3%)	27 (36.0%)	29 (25.7%)	6 (5.0%)	65 (21.7%)
**Year presented**	2002	2003	2004	2005	2006	2007	2008	2009	2010	2011	2012	2013	2014
**Patients treated (N = 1301)**	49	61	83	83	75	90	64	93	95	75	113	120	300
**Average age when treated (years)**	10.5	9.0	12.2	10.7	6.3	8.3	6.3	7.7	9.8	11.9	9.9	7.8	4.9
**Mean distance from HEAL (km)**	282.7	255.1	197.7	134.3	90.9	70.7	228.4	560.4	289.1	171.5	138.5	62.4	105.5
**N(%) relying on mercy funds**	49 (100%)	61 (100%)	40 (48.2%)	56 (67.5%)	59 (78.7%)	68 (75.6%)	56 (87.5%)	93 (100%)	93 (97.8%)	30 (40.0%)	89 (78.8%)	91 (75.8%)	244 (81.3%)
**N(%) treated by HEAL Mobile Clinic**	49 (100%)	48 (78.7%)	83 (41.0%)	32 (38.6)	13 (17.3%)	18 (20.0%)	41 (64.1%)	93 (100%)	82 (86.3%)	27 (36.0%)	29 (25.7%)	6 (5.0%)	65 (21.7%)

Over the 13-year interval, orthopedic defects were the most common (n = 519), including 458 clubfoot abnormalities (Table 2). Clubbed feet were the most commonly treated congenital abnormality (35% of all diagnoses, and 88% of all musculoskeletal abnormalities diagnosed). The next most common abnormalities were abdominal and abdominal wall defects (n = 293, 22.52%) and cleft lip/palate (n = 308, 23.67%) (Table 2).

**Table 2:** Patient Characteristics.

**Sociodemographics**			**Patients treated (total n = 1301)**	
Age (years), mean +/- SD			8.20 +/- 9.44	
Use of mercy funds (yes)			1029 (79%)	
Sex (female)			438 (34%)	
Distance from HEAL Hospital (km), mean +/- SD			178.4 +/- 249.2	
Treated by mobile clinic			537 (41%)	
**Diagnoses: N (%)**				
**Achalasia**	**ARM**	**Cataract**	**Cleft lip**	**Clubfoot**
1 (<1%)	43 (3%)	4 (<1%)	308 (24%)	458 (35%)
**Encephalocele**	**Epispadias**	**Erb’s palsy**	**Gastroschisis**	**Hemangioma**
2 (<1%)	2 (<1%)	5 (<1%)	2 (<1%)	4 (<1%)
**Hirschsprung**	**Hydrocephalus**	**Hypospadias**	**Imperforate hymen**	**Inguinal Hernia**
4 (<1%)	62 (5%)	20 (2%)	2 (<1%)	188 (15%)
**Labia Minora Coal**	**Legg Perthes**	**Morris syndr**.	**Omphalocele**	**Patella alta**
10 (1%)	6 (1%)	2 (<1%)	10 (1%)	1 (<1%)
**Phimosis**	**Polydactyly**	**Pyloric stenosis**	**Rokitansky syndr**.	**Spigelian hernia**
2 (<1%)	6 (1%)	2 (<1%)	1 (<1%)	3 (<1%)
**Spina bifida**	**Syndactyly**	**Thyroglossal canal**	**TOF**	**Umbilical hernia**
46 (4%)	24 (2%)	4 (<1%)	1 (<1%)	50 (4%)
**UPJ stenosis**	**Undescended testis**			
6 (1%)	22 (2%)			

The mortality rate for surgery was low and averaged less than 1.0% (Table 3). Mortality was highest for hydrocephalus (9.7%), while no patients died after surgery for spina bifida, hypospadias or clubfoot. Inguinal hernia repair had a 1.1% mortality rate, a result of complications immediately following surgery, typically intestinal obstructions. Other reported mortality rates observed in sub-Saharan Africa vary on a case-by-case basis. Studies report mortality associated with CM corrective surgery ranging from 0.0–5.3% in Nigeria [11,12] to as high as 9.2% in rural Tanzania [13].

**Table 3:** Number of deaths after treatment for common CMs at HEAL Africa Training Hospital, Goma, DRC (N = 1175 patients).

**Type of malformation**	**Mortality N(%)**	**Average Length of hospital stay (days)**
Clubfoot	0 (0.0)	21 (only for those we operate on which make 60% of the total clubfoot cases. The remaining 40% were treated with non-surgical methods on an ambulatory basis).
Cleft lip/palate	0 (0.0)	7
Inguinal hernia	2 (1.1)	4
Hydrocephalus	6 (9.7)	10
Umbilical hernia	0 (0.0)	2
Spina bifida	0 (0.0)	10
Anorectal malformation	0 (0.0)	10
Hypospadias	**0 (0.0)**	**10**

From 2002–2014, treated patients lived an average of 178.4 km (Table 1) away from HEAL Hospital. Only 33.1% of patients lived in Goma or the immediate surrounding areas (10 km from HEAL Hospital facilities), while 33.2% of patients lived 350 km of farther from the treatment center. When controlling for our other barriers to care, CM patients who lived farther from Goma were significantly older than those closer (p = 3.33x10^-6^) (Table 4). Even when included as an interaction variable with distance, surgery location (HEAL Hospital or Mobile Clinic) had no effect on our model.

**Table 4:** R output summarizing the significant (p = 0.05) predictors of age at time of surgery in our best-fit model. Individual significant diagnoses were not included in this table but can be found in S2 Table. The model had AIC of 774.0, and an adjusted R^2^ value of 0.41.

**Coefficients**:	**Estimate**	**Std. Error**	**t-value**	**p-value**
**Intercept**	47.2295493	21.4547217	2.201	0.027892
**Distance**	0.0007853	0.0001681	4.670	3.33x10^-6^
**Year**	-0.0223010	0.0106797	-2.088	0.036984
**Sex (Male)**	-1.6347432	0.4971965	-3.288	0.001037
Payment (mercy funds)*Sex (Male)	0.7331404	0.2577971	2.844	0.004529

As expected, due to the immense poverty in the area, the majority of patients (79.1%) relied on mercy funds to pay for treatment. When running our series of initial simple regressions, we found that younger patients were significantly more likely to rely on mercy funds to cover treatment costs (p<0.001), and that gender was not a significant predictor of age at time of treatment. However, after observing a strong correlation between gender and use of mercy funds, we decided to include an interaction term into our complete multivariate regression model to account for suspected autocorrelation. This interaction term would be included in out best fit model, where it proved to be a significant predictor (p = 4.53x10^-3^) of age at time of treatment, with females being significantly more frequent users of mercy funds and thus significantly older at time of treatment. With the inclusion of this interaction term, gender alone was also identified a significant predictor (p = 1.04x10^-3^) of age at time of treatment (Table 4), with males undergoing surgery at an earlier age than females.

## Discussion

During the 1990s, there were limited to no options for individuals who needed surgical correction of any CMs in North Kivu Province and the surrounding area. As a result, belief spread that CMs were untreatable. In some cultures, such as Hausa and Fulani, CMs are considered the ''Will of God'' against which nothing should be done [14], while in rarer, more extreme cases, babies born with congenital anomalies are considered curses, and killed [14]. Fortunately, with the increasing availability of medical and surgical interventions to correct CMs, community awareness is growing. The number of successful surgical corrections of CMs has increased, as reflected by the increase of treated cases from 2002 to 2014 (Table 1), particularly in the last three years. During that time, HEAL Hospital has also been able to successfully treat patients at a significantly earlier age, thus avoiding major complications later in life (Fig 1, Tables 1 and 4). Increased awareness has been buoyed by the fact that successfully treated individuals hailing from more remote areas have been returning home and informing other potential patients that are living in the jungle that treatment is safe and effective.

Though more individuals are being treated by HEAL, we found that distance is still the most significant predictor of delayed correction (Table 4). Accordingly, we believe that distance is the greatest barrier to CM treatment in the eastern DRC, with patients and surgeons travelling an average of 178.4 km to undergo/perform surgery, sometimes even by plane. With limited infrastructure in rural areas, travel is often difficult, although not impossible as demonstrated by our data. HEAL’s Mobile Clinic has treated patients as far away as Karawa, Equator Province, approximately 1420km from Goma (20 individuals in 2009). In addition to poor infrastructure, another issue complicating travel to and from HEAL is the continued armed conflict in the region. Despite the formal end to the war in the DRC, the prolonged menace of armed rebels in the eastern DRC countryside is likely still intensifying this barrier. Accordingly, we demonstrate that further funding is desperately required from the National Government or International non-profit agencies to enable continued treatment of CMs to rural residents in the DRC, whether it is via the implementation of more mobile clinics or by the construction of additional regional surgical centers.

The patterns observed of the most common CMs in the DRC are dissimilar to a report from Nigeria [7], including 310 babies, where the most commonly diagnosed types of malformations involved the gastro-intestinal system (140 cases; 45.2%) and the central nervous system (75 cases; 24.2%), which is strikingly different from the types of diagnoses observed in our sample. However, our overall male to female ratio of roughly 2:1 was similar to that reported in Nigeria [15].

The sex ratio and age range observed in this study are also dissimilar to reports from other African countries (with the exception of the similar sex ratio compared to Nigeria, see above). In Ghana, out of 74 new cases of cleft lip and palate observed, 33 of the patients were identified as male and 41 as female. Their ages ranged from one day to 21 years, and they presented at a significantly lower average age of 10 months [16] compared to our average age of 8.2 years. The age and distribution in Ghana is also similar to the findings from a Kenyan report, where significantly more women were treated [10]. Only 33.7% of our sample was female, and they had to travel significantly (p = 1.29x10^-6^ per one way ANOVA) farther for care (70.5 km farther then males on average). Females also had a heavier reliance on mercy funds than males (89.0%, as opposed to ~74% of males). This could be due to the propensity of families in DRC to spend their own money on treating male members, or perhaps the smaller Ghanaian and Kenyan sample size may have biased their sex ratio comparisons. Congenital anomalies are most commonly seen in low-income families who have limited access to health care [16,17]. These factors explain in part the delayed patient presentation found in this study, as well as the high reliance on mercy funds to cover treatment, particularly in the case for women.

A comparison of patients age at time of consultation with congenital anomalies in this study and elsewhere suggests that the delayed ages of presentation at HEAL Africa Hospital are among the highest worldwide [16]. This may be explained by the DRC’s poor health system, lack of awareness initiatives, the lack of a plastic surgery center in the country, the absence of roads, etc. To our knowledge, there are two plastic surgeons [6] in the whole of the DRC (around 74 million people) (Table 5). Delayed consultation combined with lack of specialized care is a major health challenge faced in DRC, though based on our findings, the average age at time of treatment is significantly decreasing, from approximately 12 years in 2004 to less than 5 years in 2014 (Fig 1; Table 1).

**Table 5:** Number of surgeons in the DRC, demonstrating the shortage of specialists in the country. DRC is ranked 187th out of 187 countries on the planet as per the Human Development Index calculated by the United Nations.

**Specialties**	**Nb**	**Nb of surgeons/million**
**Surgeons (general)**	75	1.01
**Plastic surgeons**	2	0.03
**Orthopedic surgeons**	10	0.14
**Neurosurgeon**	4	0.05
**Pediatric surgeon**	8	0.11
**Thoracic surgeon**	2	0.03
**Urologist**	2	0.03

Anorectal malformations were reported to be very common in other African countries like Uganda and Nigeria [9,7]. This is not the case in our setting. This difference in diagnosis/presentation is likely due to the inability to access care in time to correct the defect in DRC, thus many die before reaching the health facility. Those born in the remote jungle without access to surgeons and radiologists, as well as not having money available to cover fees, often stay home untreated until death. Lastly, there are cultural beliefs that consider CMs as curses, and patients with this condition will often expire under the care of traditional doctors.

Unfortunately, we did not capture the differences in outcomes allowing the value of early identification and treatment and how this minimizes disabilities and promotes improved outcomes, something that will be monitored going forward. Neither did we record the complications/number of surgeries/length of hospital stays for neglected malformations, which were only available for a small percentage of the study population. These data could be used for future studies.

## Conclusion

Though residents of the eastern DRC diagnosed with CMs have been better able to successfully grapple with certain barriers to care in recent years, as indicated by the decrease in age of the patients treated over our thirteen-year time frame, travel and cost are still very serious obstacles to corrective treatment. This study found that over that interval, patients are being treated by HEAL Hospital significantly earlier in life. We find that delayed surgery can be significantly predicted by a patient’s distance from hospital, and that women in particular have been relying on mercy funds to cover the cost of travel and treatment, thus further delaying care (Table 4). With 89% of women and girls, and over 81% of all patients, requiring mercy funds to cover the cost of surgery in 2014, we conclude that the continued need for aid is still substantial. Of all the CMs diagnosed at HEAL Hospital, clubfoot was the most commonly treated, which was expected as HEAL Hospital is the National Centre for Clubfoot Care. Some of the other more prevalent (n>35) CMs diagnosed included: cleft lip, inguinal hernia, hydrocephaly, umbilical hernia, and spina bifida.

Although patients have been undergoing corrective surgery at significantly younger ages over the 13-year study interval, the average age at time of presentation to HEAL Africa Hospital, Goma, DRC or its Mobile Clinic is still 4.9 years for 2014, which is high for sub-Saharan Africa. Taking this number into account, there is a clear need for the construction of additional regional surgery centers in an effort to provide localized care for patients in rural DRC, directly combatting the most significant barrier to care. Furthermore, to ensure earlier treatment in rural aras, we recommend continued large scale community education/sensitization campaigns informing the public about the treatment options available, early consultation, availability of money to cover care/travel, and success rate of corrective procedures [18,19,20,21]. Over time we hope that we can educate individuals that congenital abnormalities are not a divine curse, and encourage corrective treatment rather than other, often more morbid alternatives. In addition to disseminating information regarding treatment options and efficacy, going forward, we intend to investigate other barriers to care, as well as how the serum folate levels of girls in regards to reproductive health, factors of delayed consultation, and cultural beliefs about CMs in the regions where consultation is hindered by accessibility.

## Supporting Information

S1 Table Complete CM dataset utilized in this study. (CSV)

S2 Table Complete output of best-fit model including individual significant diagnoses. (CSV)
